# Multi-organ single-cell analysis of preferential expression of CAKUT genes

**DOI:** 10.1186/s12882-026-05003-y

**Published:** 2026-04-27

**Authors:** Mara Pflugfelder, Antonia Eberts, Vera Koch, Illya Martynov, Carlo Maj, Guido Seitz, Johannes Schumacher, Mohammad Reza Vahdad, Pouria Dasmeh

**Affiliations:** 1 Center for Human Genetics, Philipps University of Marburg, Marburg, Germany; 2 Department of Pediatric Surgery and Pediatric Urology, University Hospital Giessen-Marburg, Marburg, Germany

**Keywords:** CAKUT, Fetal kidney, Development, Single-cell transcriptomics, Cell Types

## Abstract

**Background:**

Congenital anomalies of the kidney and urinary tract (CAKUT) comprise a heterogeneous group of developmental disorders with diverse genetic etiologies. However, it remains unclear whether these genetic factors converge on shared cellular programs across development and tissues.

**Methods:**

We analyzed the expression of 91 curated CAKUT-associated genes across publicly available single-cell RNA sequencing datasets from human fetal and adult kidney, ureter, and bladder. These data were complemented by early embryonic transcriptomic profiles to characterize temporal expression dynamics and cell-type specificity.

**Results:**

During kidney development, key CAKUT genes, including *EYA1*,* SIX1*,* PAX2*, and *FOXC1*, showed strong preferential expression in mesangial and mesonephric nephron tubule epithelial cells, highlighting early roles in ureteric bud induction and branching morphogenesis. Temporal analysis identified two distinct expression patterns: an early-peak group (*EYA1*,* SIX1*,* SIX2*,* PAX2*,* ITGA8*) with maximal expression during early nephrogenesis followed by decline, and a late-rise group, including *MUC1*, with increasing expression toward adult stages. Across tissues, CAKUT genes exhibited a conserved enrichment in stromal and mesenchymal cell populations.

**Conclusions:**

Our findings reveal a shared stromal–mesenchymal gene expression signature underlying CAKUT pathogenesis. These results suggest that diverse genetic perturbations may converge on early mesodermal lineage programs that are critical for ureteric bud formation and kidney patterning, providing a unifying cellular framework for understanding CAKUT.

**Supplementary Information:**

The online version contains supplementary material available at 10.1186/s12882-026-05003-y.

## Introduction

Congenital anomalies of the kidney and urinary tract (CAKUT) represents a broad spectrum of structural and functional malformations, ranging from severe defects such as renal agenesis to milder conditions like vesicoureteral reflux [1, 2]. CAKUT is among the most common congenital disorders, accounting for up to 20% of all prenatally detected birth defects during routine second-trimester fetal anatomical surveys [3–5]. It also represents a major cause of pediatric kidney failure, contributing to approximately 40% of end-stage renal disease cases within the first three decades of life [6]. Congenital renal malformations are defined macroscopically by changes in kidney size, shape, position or microscopically by a reduced number of nephrons and/or abnormal histology. Depending on the severity of the CACUT, the deformity can usually be detected antenatally after urine production between the 16th and 18th week of pregnancy with an accuracy of 65–85%. If the changes occur in the later stages of pregnancy, the distal areas of the urinary tract are usually affected, which usually lead to an obstruction.

CAKUT reflects a heterogeneous genetic architecture that spans rare coding variants, structural alterations, and polygenic contributions. More than 50–60 genes have been implicated in monogenic forms, together accounting for roughly 10–20% of isolated cases, while pathogenic copy number variants explain an additional 4–11% [2, 7, 8]. Evidence for common-variant involvement is emerging, though current GWAS and candidate-gene studies (e.g., in *GREM1*, *EYA1*, *ROBO2*, *UPK3A*) remain underpowered, suggesting that polygenic architecture exists but is incompletely charted [9, 10]. What remains strikingly underexplored is when and where genetic changes contribute to CAKUT. Like other congenital malformations, CAKUT unfolds along two developmental axes: a *spatial* axis, defining the cell types and tissues in which risk genes act, and a *temporal* axis, capturing the developmental windows when their effects are most critical.

On a spatial level, the key question is where CAKUT genes show their highest preferential expression within the urinary tract. The kidney develops through tightly regulated morphogenetic programs driven by reciprocal interactions between the ureteric bud and the metanephric mesenchyme [11–13]. This involves multiple cell types, including nephron progenitors, ureteric epithelial cells, stromal cells, podocytes, and tubular epithelia, each of which contributes critically to organ structure and function. Perturbation of CAKUT genes may therefore disrupt specific compartments, particularly at epithelial–mesenchymal interfaces that coordinate branching morphogenesis and nephron formation. Yet, CAKUT is not confined to the kidney alone. Developmental disruptions can also occur in adjacent tissues of the urinary tract, including the ureter, bladder, and urethra, which share a developmental continuum with the intermediate mesoderm. For instance, abnormal signaling in ureteric mesenchyme can lead to ureteral obstruction or vesicoureteral reflux, while defects in smooth muscle or fibroblast lineages may underlie bladder dysfunction and urethral anomalies [14, 15]. Thus, mapping the spatial expression of CAKUT genes across the entire urinary tract is essential for pinpointing the cellular compartments most vulnerable to genetic perturbations and for understanding how organ-specific anomalies arise within a shared developmental framework.

On a temporal level, a central unresolved issue is when CAKUT risk is specified during human development. Most CAKUT cases trace to early perturbations of ureteric bud and mesenchyme interactions, which in humans begin around gestational week 4. This is a developmental window through which the ureteric bud emerges from the nephric duct and invades the metanephric mesenchyme [1, 2, 16]. Disruption during this critical window is likely to shape CAKUT. Single-cell transcriptomics allows inference of such timing by localizing disease-associated genes to stage-defined lineages, from intermediate mesoderm to stromal progenitors and later epithelial compartments.

Recent advances in single-cell transcriptomics provide a principled framework for addressing both the spatial and temporal dimensions of congenital disease. Previous works have demonstrated that disease-associated genes tend to be preferentially expressed in the cell populations they perturb, and that mapping disease gene sets onto single-cell expression landscapes enables systematic prioritization of disease-relevant cell types [17–20]. In particular, single-cell analyses of the human fetal kidney have shown that scRNA-seq faithfully captures key developmental programs relevant to congenital renal disorders [21], while integrative single-cell strategies combining disease gene sets or genetic information with cell-resolved expression profiles have been validated across a range of complex and developmental conditions [18, 22]. Together, these studies establish single-cell transcriptomics as a robust approach for resolving the cellular and developmental contexts in which human disease risk is specified [22].

Here, and to resolve the spatiotemporal context of CAKUT, we examined the single-cell preferential expression patterns of genes associated with CAKUT across diverse cell types using the large-scale and high-resolution single-cell atlases of the developing kidney, as well as in two major components of the urinary tract, the ureter and bladder. While non-genetic and environmental factors, particularly during critical periods of prenatal development, are known to influence CAKUT [1], our analysis focuses on the genetic contributors to the disease. Our approach builds on the premise that disease-relevant genes must be expressed in the affected cell types, a principle widely used in gene and cell-type prioritization based on genetic data [17, 20, 23, 24]. Our analysis shows that CAKUT genes display consistent preferential expression in stromal lineages across the urinary tract—peaking in mesangial populations in the fetal kidney and in mural/perivascular, vascular smooth muscle, and fibroblast lineages in the adult kidney, ureter, and bladder. Early embryonic expression localizes to intermediate-mesoderm–derived populations, placing this signal prior to organogenesis and defining a stromal cellular context for CAKUT.

## Methods

### Data compilation

To compile a comprehensive list of genes implicated in CAKUT, we conducted a literature review and identified 91 genes with strong evidence for association with CAKUT (see Table S1). We curated this set of genes, hereafter referred to as CAKUT genes, based on two comprehensive reviews of CAKUT genetics (Kolvenbach et al., 2023 [2]; Talati et al., 2019 [25]). We included genes reported as causative or strongly implicated in human CAKUT phenotypes, with supporting evidence from monogenic disease studies, including pathogenic or likely pathogenic variants identified in human cohorts. We excluded genes lacking functional validation, copy-number variants without gene-level resolution, and genes supported only by animal-model evidence without confirmed relevance in humans. Table S1 summarizes the curated genes together with the source reference, type of genetic evidence (where available from ClinGen [26]), mode of inheritance, associated phenotype, and gene identifiers, including HGNC symbol and Ensembl ID.

To investigate the expression patterns, we integrated multiple single-cell and single-nucleus transcriptomic datasets spanning distinct regions and developmental stages of the urinary tract. The Hochane et al. [27] dataset comprises single-cell transcriptomes from ~ 18,000 fetal kidney cells across five developmental stages (gestational weeks 7–16). Each cell was annotated to one of 22 cell types, including several subpopulations of nephron progenitor cells. The Lake et al. [28] dataset includes over 400,000 kidney cells profiled using single-cell and single-nucleus RNA sequencing from 45 healthy donors and 48 patients. These were clustered into 51 annotated cell types. The dataset of Fink et al. [29] contains 30,942 ureteral cells collected from ten adult patients, analyzed using single-cell RNA sequencing (scRNA-seq) and spatial transcriptomics. The atlas of Santo et al. [30] encompasses 29,834 bladder cells, including 22,231 single cells and 7,603 single nuclei, sampled from four anatomical regions of the human bladder. To complement these datasets and address potential inconsistencies in cell-type annotation across studies, we additionally analyzed the *Tabula Sapiens* atlas [31], a harmonized multi-organ single-cell resource with standardized cell-type annotations and a unified processing framework. We restricted this dataset to the available urinary tract tissues (kidney and bladder) and used it as an independent reference to validate the robustness of our findings across studies with differing annotation schemes.

To ensure comparability across datasets generated using different technologies (scRNA-seq and snRNA-seq), we reprocessed all expression matrices using a uniform preprocessing pipeline implemented in Scanpy (v1.5.0). Specifically, we applied standard quality-control filtering to remove low-quality cells and rarely detected genes, followed by library-size normalization to 10,000 counts per cell and log-transformation (log1p). The resulting log-normalized expression matrices were used for downstream enrichment analyses.

All primary single-cell datasets analyzed in this study are publicly available with no access restrictions. Accession numbers and permanent dataset identifiers include CELLxGENE collections bcb61471-2a44-4d00-a0af-ff085512674c, 981b0c87-76d0-4edf-b201 58c52b5cd2f0, 0e54d4de-44f0-4d50-8649-b5c2bbe8f5d1, and af286675-7167-4816-a907-c72d41b73d37, as well as the Gene Expression Omnibus (GEO) dataset GSE157329. Full dataset accessibility, direct links, and processing details are provided on the project GitHub repository (https://github.com/dasmeh/CAKUT). No controlled-access datasets were used, and no additional permissions were required to access the data.

### Identification of disease relevant cell types

To identify cell types relevant to CAKUT pathogenesis, we applied the single-cell Disease Relevance Score (scDRS) method, which integrates single-cell RNA-seq data with polygenic risk information at the single-cell level [17]. This approach quantifies a disease relevance score for each cell, independent of prior cell-type annotations, by assessing the relative overexpression of disease-associated genes. Specifically, scDRS evaluates whether a given cell exhibits excess expression of genes implicated in a disease, using 1,000 randomly matched control gene sets to assess statistical significance.

To extend this framework from cell-level prioritization to gene-level interpretation, we next examined how individual gene expression patterns track disease relevance scores across cells. For each gene, we quantified the correlation between its expression level and the scDRS-derived disease scores across all cells. Genes whose expression is highest in cells with elevated disease relevance scores are thus identified as co-varying with disease-associated transcriptional programs.

### Cell-type-specific expression of CAKUT genes

To determine whether each CAKUT gene exhibited cell-type-specific expression across the 22 annotated fetal kidney cell types, we performed the Kruskal–Wallis test for each gene. This non-parametric test evaluates whether the distribution of expression values differs significantly between cell types. In brief, assuming the following notations: For each gene g ∈ G, we test the null and alternative hypotheses as H_0_, where the distribution of X_g, c₁_, X_g, c₂_, …, X_g, c₂₂_ are the same (indicating no cell-type specificity), and H_a_, where at least one cell type c_i_ has a different expression distribution for gene g, respectively. We apply the Kruskal–Wallis test to compute a p-value for each gene *g*. To correct for multiple testing, the Benjamini–Hochberg procedure is applied across all p-values. Genes with FDR-adjusted p-values less than α (e.g., α = 0.05) are considered significantly cell-type-specific.

## Results

### Compilation of CAKUT genes and single-cell transcriptomics data sets

In this study, we investigated the preferential expression of 91 genes associated with CAKUT, systematically curated from human genetic studies and annotated for strength of evidence using ClinGen classifications (e.g., Definitive, Moderate), with additional support from functional and animal model studies (see Methods, and Table S1). Although temporally matched and cell type–harmonized single-cell datasets and atlases are rare, we performed our analyses across multiple single-cell datasets to ensure consistency, replication, and the ability to compare embryonic and fetal gene expression patterns with those in adult kidney cells (Table 1).

We first investigated the expression of CAKUT genes among different cell types of the fetal kidney cells using the single-cell atlas of Hochane et al. [27], which includes approximately 18,000 renal cells, classified into 22 distinct cell types across five developmental time points, ranging from day 48–51 post-fertilization (corresponding to the stage HsapDv:0000026) to week 16 of gestation (HsapDv:0000053). This developmental window is particularly relevant, as it overlaps with the period during which CAKUT can first be detected. The fetal kidneys begin to be visualized by ultrasound between weeks 12 and 15 of pregnancy, and CAKUT is typically diagnosed between weeks 16 and 20 [32]. To extend our analysis into postnatal stages, we also incorporated adult single-cell transcriptomic datasets from the kidney, particularly from human cell atlas (Lake et al. [28]), ureter (Fink et al. [29]), and bladder (Santo et al. [30]), encompassing a total of 271,148 cells (Table 1).

### CAKUT genes exhibit cell-type-specific expression during human kidney development

**Fig. 1:**
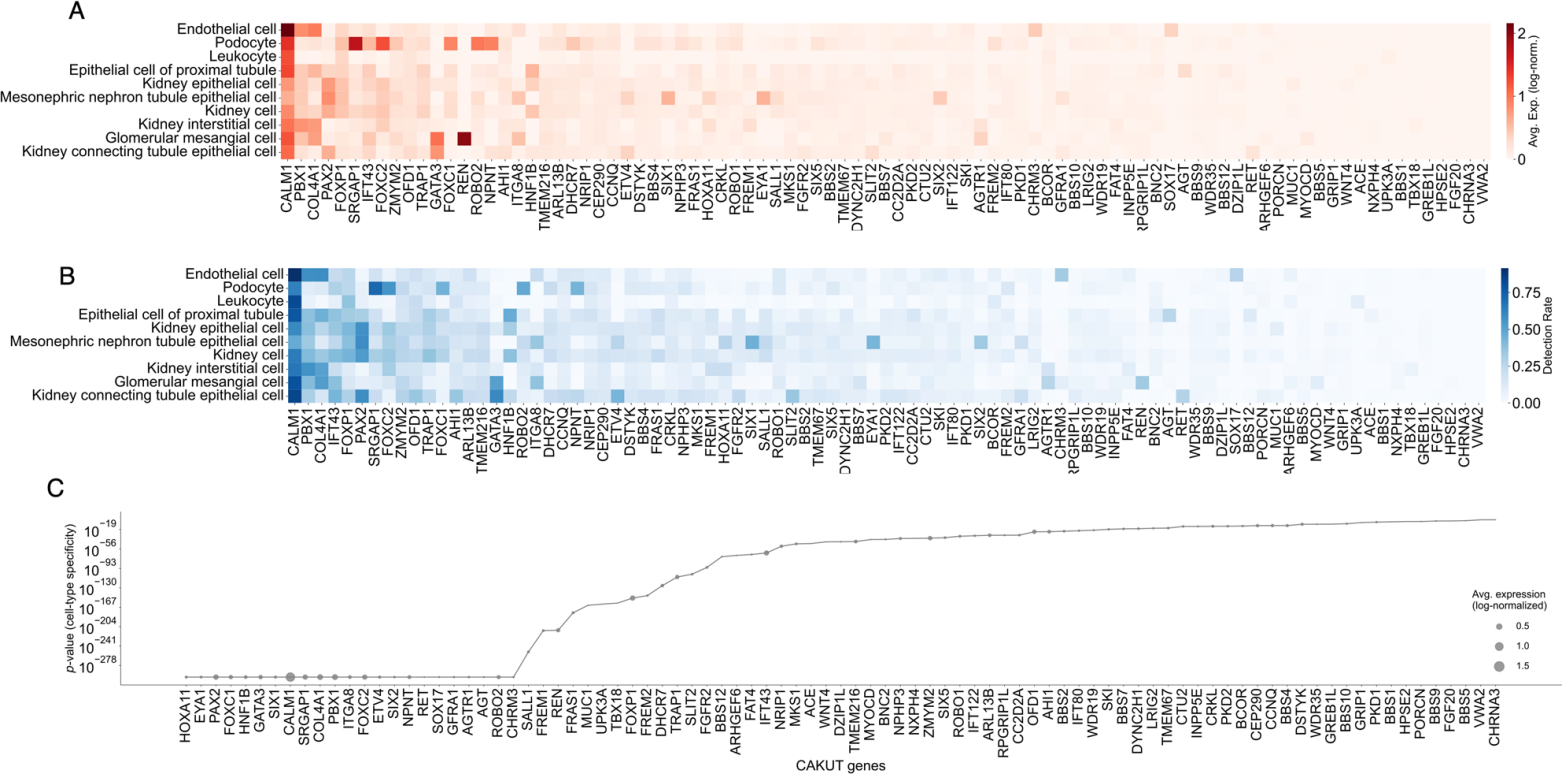
Cell-type-specific expression of CAKUT genes in the human fetal kidney cells. (**A**) Heatmap showing the average expression of 91 CAKUT genes across 22 fetal kidney cell types. Expression values are log-normalized and averaged per cell type. (**B**) Detection rate heatmap for the same genes, representing the proportion of cells within each cell type in which the gene is detectably expressed (non-zero expression). Genes and cell types are ordered consistently with panel A. (**C**) Statistical evaluation of cell-type specificity for each CAKUT gene. Each gene is represented as a point on a line plot, where the y-axis shows the p-value from the Kruskal–Wallis test (log scale), and the size of each point reflects the gene’s average expression level (log-normalized). A subset of genes, including *HOXA11*, *EYA1*, *PAX2*, *FOXC1*, *HNF1B*, *GATA3*, *SIX1*, and *ITGA8*, exhibited extremely significant cell-type-specific expression, with p-values reaching the computational minimum (≤ 1 × 10^− 300^)

We first quantified the expression landscape of CAKUT genes across the 22 distinct kidney cell types annotated in the single-cell atlas of Hochane et al. [27] (see Table S2). For each gene, we calculated the average expression level as the mean of log-normalized, pseudocount-corrected values across cells within each annotated cell type (Methods; Fig. 1A). To complement this, we computed the detection rate, defined as the proportion of cells in which the gene was detectably expressed (i.e., non-zero expression) within each cell type (Fig. 1B, Table S3). From the figures, we observe a substantial heterogeneity in both average expression and detection rates of CAKUT genes. While a few genes had a broad expression across the cell types (seen on the left side of the heatmaps), most genes were expressed specifically in distinct cells (as seen toward the right side of the heatmaps).

To determine whether each CAKUT gene exhibited cell-type-specific expression or was more broadly expressed across kidney cell populations, we performed a statistical comparison of gene expression distributions across all 22 annotated fetal kidney cell types. For each gene, we used the Kruskal–Wallis test and evaluated whether the observed expression of a gene was significantly concentrated in one or a few cell types rather than uniformly distributed (see Methods). To account for multiple testing across all genes, we applied the Benjamini–Hochberg procedure to control the false discovery rate (FDR).

As shown in Fig. 1C, all CAKUT genes except two, *CHRNA3* and *VWA2*, displayed statistically significant cell-type-specific expression (FDR < 0.05), indicating widespread specificity during human kidney development. For a subset of these genes—including *HOXA11*, *EYA1*, *PAX2*, *FOXC1*, *HNF1B*, *GATA3*, *SIX1*, and *ITGA8*—the *p*-values reached the computational minimum (≤ 1e-300), reflecting exceptionally strong evidence for cell-type-specific expression. Notably, many of these genes are well-established in the etiology of CAKUT and are among the most frequently implicated in affected patients [2]. Motivated by these findings, we next sought to systematically rank cell types based on their expression profiles of CAKUT genes.

### Cell type specific expression of CAKUT genes in fetal kidney cells

To identify the fetal kidney cell types with the strongest expression of CAKUT genes, we employed two complementary approaches: (1) a gene-wise analysis assessing cell-type specificity for individual genes, and (2) a gene set-based enrichment analysis evaluating collective expression across cell types.

**Fig. 2:**
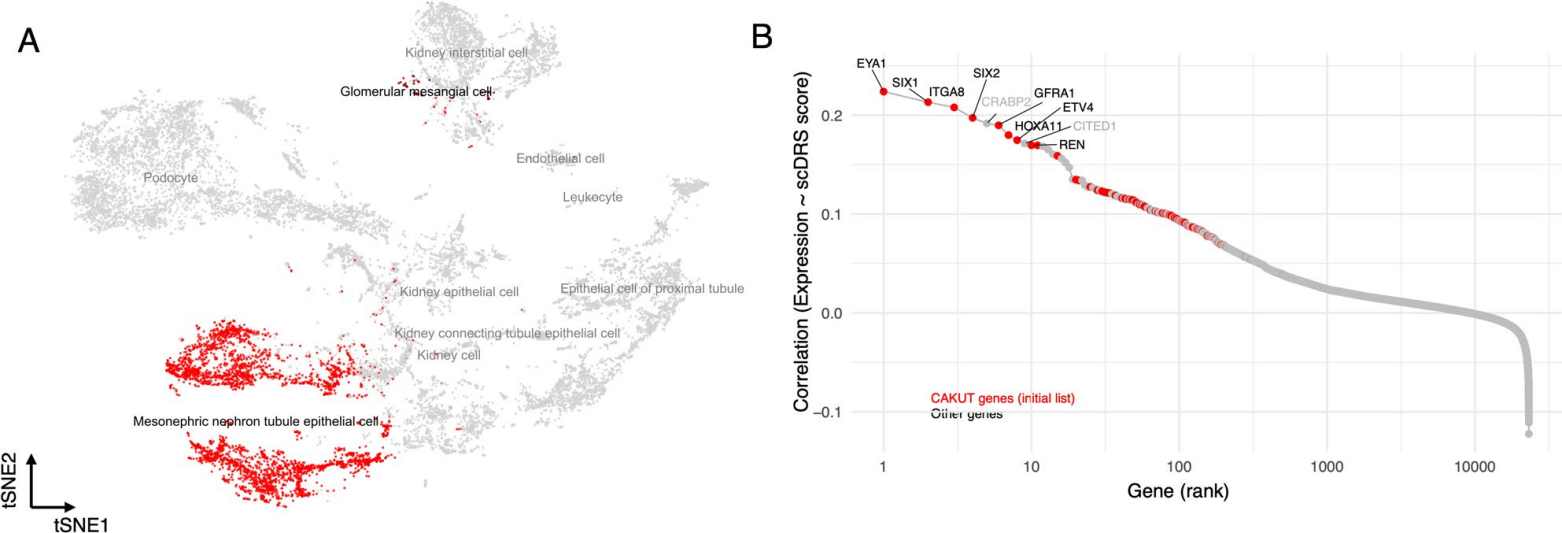
Preferential expression of CAKUT genes in fetal kidney cell types. (**A**) *t*-SNE projection of embryonic kidney single-cell transcriptomes showing the spatial distribution of major fetal cell types from the atlas of Hochane et al. Cell types that show statistically significant enrichment of CAKUT gene expression (based on scDRS analysis, *p* < 0.05) are highlighted in red. Non-significant cell types are shown in gray. (**B**) Correlation of gene expression across embryonic kidney cells with CAKUT disease scores derived from scDRS. Each dot represents one gene ranked by its correlation with the CAKUT scDRS score. CAKUT genes are highlighted in red, and non-CAKUT genes are shown in gray. Several top-ranking genes are labeled, including well-known CAKUT genes such as *EYA1*, *PAX2*, *HNF1B*, and *SIX1*

In the first approach, we calculated the average log-normalized expression of each CAKUT gene across all annotated kidney cell types. For each gene, we identified the cell type with the highest mean expression, defined as its top-expressing cell type (see Methods). We then quantified how frequently each cell type was identified as the top-expressing cell type across the CAKUT gene set and compared these observed counts to the expected frequencies based on the background distribution of cell types in the atlas of Hochane et al. [27]. We used a chi-squared test to assess significance, and the observed-to-expected ratio was used to quantify enrichment. We observed a highly significant deviation from the expected distribution (Chi-squared test, *p* < 2.2 × 10^–16^), indicating strong cell-type-specific expression patterns. Notably, glomerular mesangial cells showed the strongest overrepresentation, followed by connecting tubule epithelial cells (Fig. 2A), suggesting that these two cell types most frequently exhibit preferential expression of CAKUT genes.

In the second approach, we treated the CAKUT genes as a single set and tested for preferential expression of this gene set at the cell type level using single-cell disease relevant score (scDRS) methodology, an approach that identifies statistically significant enrichment of gene set expression in single-cell RNA-seq data while accounting for gene expression variability and technical noise (see Methods) [33]. We found glomerular mesangial cells (adjusted *p* = 0.0009, permutation test) as well as mesonephric nephron tubule epithelial cell (adjusted *p* = 0.0069, permutation test) to show a significant preferential expression of CAKUT genes (Table S4). Notably, a key distinction between these two populations was that heterogeneity of preferential expression was only significant in mesonephric nephron tubule epithelial cells (*p* = 0.011; scDRS-based heterogeneity test using permutation-derived null distribution), suggesting that this cell type likely contains subpopulations with markedly elevated CAKUT gene expression. By contrast, mature nephron compartments, including proximal tubule epithelia, podocytes, and collecting duct cells, showed little or no enrichment (all *p* > 0.2).

**Table 1:** Single-cell datasets used in this study to evaluate the preferential expression of CAKUT genes, along with the statistical significance and heterogeneity of their enrichment as determined by the scDRS method

Dataset	Cell types	*p*-value (association)	*p*-value (heterogeneity)	Reference
Fetal kidney cells	Glomerular mesangial cells	0.000999	0.8101898	(Hochane M, 2019 [27])
	Mesonephric nephron tubule epithelial cells	0.006993	0.01198801	
Adult kidney cells	Mural cells	0.000999001	0.002997003	(Lake BB, 2023 [28])
	Vascular associated smooth muscle cells	0.003996004	0.34465533	
	Kidney collecting duct principal cells	0.004995005	0.000999001	
	Kidney loop of Henle thin descending limb epithelial cells	0.004995005	0.0989011	
	Glomerular capillary endothelial cells	0.012987013	0.008991009	
	Kidney inner medulla collecting duct epithelial cells	0.016983017	0.64735264	
	Kidney arterial blood vessel cells	0.02097902	0.20779221	
Adult ureter cells	Smooth Muscle cells	0.000999	0.000999	(Fink EE, 2022)
	Fibroblasts	0.000999	0.896104	
	Fibroblasts	0.000999	0.014985	
	Endothelial cells	0.008991	0.324675	
Adult bladder cells	Peri-urothelial fibroblasts	0.005994	0.005994	(Santo B, 2024)
	General smooth muscle cells	0.007992	0.000999	
	Intra-muscular fibroblasts	0.007992	0.001998	
	CXCL14-hi fibroblast	0.018981	0.001998	
	Vascular smooth muscle cells	0.035964	0.4195804	

Although scDRS accounts for differences in baseline gene expression levels through matched control gene sets, we further considered the possibility that our results could be influenced by an additional confounder, namely gene length, which can affect the probability of transcript detection in single-cell data. We therefore generated 1,000 random control gene sets matched simultaneously for both gene expression level and gene length, using gene annotations based on the GRCh38 genome assembly. Specifically, for each CAKUT gene, control genes were sampled from the same expression and length bins, ensuring comparable detection probability. We then recalculated the preferential expression score for each kidney cell type using these matched control gene sets to derive an empirical null distribution, and quantified significance using empirical p values and z scores. This analysis confirmed that the preferential expression of CAKUT genes remained stronger than expected under the matched null model in key fetal kidney populations (Table S5). In particular, glomerular mesangial cells showed the strongest enrichment (observed score = 0.0088, null mean = -0.0258, z = 3.30, empirical *p* = 0.002), followed by mesonephric nephron tubule epithelial cells (observed score = 0.0055, null mean = -0.0125, z = 2.22, empirical *p* = 0.008). In contrast, other cell types did not show evidence of enrichment beyond background expectations after matching for both expression level and gene length. These results indicate that the observed signal is unlikely to be explained simply by the high detectability of longer or more highly expressed genes, and instead supports genuine preferential expression of CAKUT genes in specific developmental kidney cell populations.

### Key gene drivers of cell-type–specific CAKUT expression in the developing kidney

The preferential expression of CAKUT-associated genes in specific embryonic kidney cell types is driven by the contribution of individual genes. Identifying which genes contribute most strongly to this signal can help prioritize candidates for follow-up studies. This is often quantified by ranking genes whose expression levels correlate most strongly with the preferential expression signal across cell types [34]. Prior studies have shown that genes at the top of such rankings play a substantial role in the disease etiology and are significantly enriched for known drug targets [17]. To identify which genes are most responsible for the observed enrichment, we computed the Pearson correlation between the expression of 23,166 human genes and the scDRS-derived disease scores across individual cells (Fig. 2B, Table S6). This approach allows us to rank genes based on how closely their expression patterns track with the overall CAKUT signal.

Several established CAKUT genes—such as *EYA1*,* PAX2*,* HNF1B*, and *SIX1*—ranked among the top contributors, consistent with their well-established roles in kidney development and CAKUT pathogenesis. While this enrichment is expected given the developmental origin of CAKUT, it provides an important internal validation that our analytical framework captures biologically relevant cell-type–specific programs and, importantly, enables us to move beyond individual gene-level associations toward identifying the precise cellular contexts in which these disease-relevant transcriptional programs operate. In addition, among the top-ranked genes showing the strongest concordance between expression patterns and disease relevance scores, we identified *CRABP2* and *CITED1*, which were not part of our curated CAKUT gene list. *CRABP2* encodes a cellular retinoic acid-binding protein that modulates retinoic acid signaling, a pathway essential for kidney morphogenesis [35]. CITED1 encodes a transcriptional co-activator expressed in nephron progenitor cells during kidney development [36]. These observations are further explored in the Discussion, particularly in the context of the interpretation of scDRS-based associations. We next turn to a comparative analysis of cell type–specific expression patterns across embryonic and adult tissues.

### Cell type specific expression of CAKUT genes in adult kidney cells

Next, we sought to prioritize CAKUT-associated genes based on their developmental contribution—distinguishing those that play a predominant role during embryonic kidney formation from those with sustained or emerging relevance in the adult organ. To this end, we analyzed adult kidney tissue using the single-cell RNA-seq atlas generated by Lake et al. [28]. This atlas profiles approximately 400,000 cells across 45 healthy and 48 diseased kidneys, covering 51 distinct kidney cell types at high resolution. To focus on physiological expression patterns, we restricted our analysis to cells derived from healthy individuals. This allowed us to characterize baseline cell-type-specific expression of CAKUT genes without potential confounding effects of disease-related transcriptional changes.

We then compared these adult expression patterns with those observed during fetal development to assess whether cell types enriched for CAKUT gene expression in the developing kidney retain this signature into adulthood. Among adult kidney cell types, CAKUT-associated genes showed significant preferential expression in several distinct populations. We observed the strongest enrichment in mural cells (*p* = 0.00099), followed by vascular-associated smooth muscle cells (*p* = 0.00399), collecting duct principal cells (*p* = 0.00499), thin descending limb epithelial cells of the loop of Henle (*p* = 0.00499), glomerular capillary endothelial cells (*p* = 0.01299), inner medulla collecting duct epithelial cells (*p* = 0.01698), and arterial blood vessel cells (*p* = 0.02098) (Table 1).

**Fig. 3:**
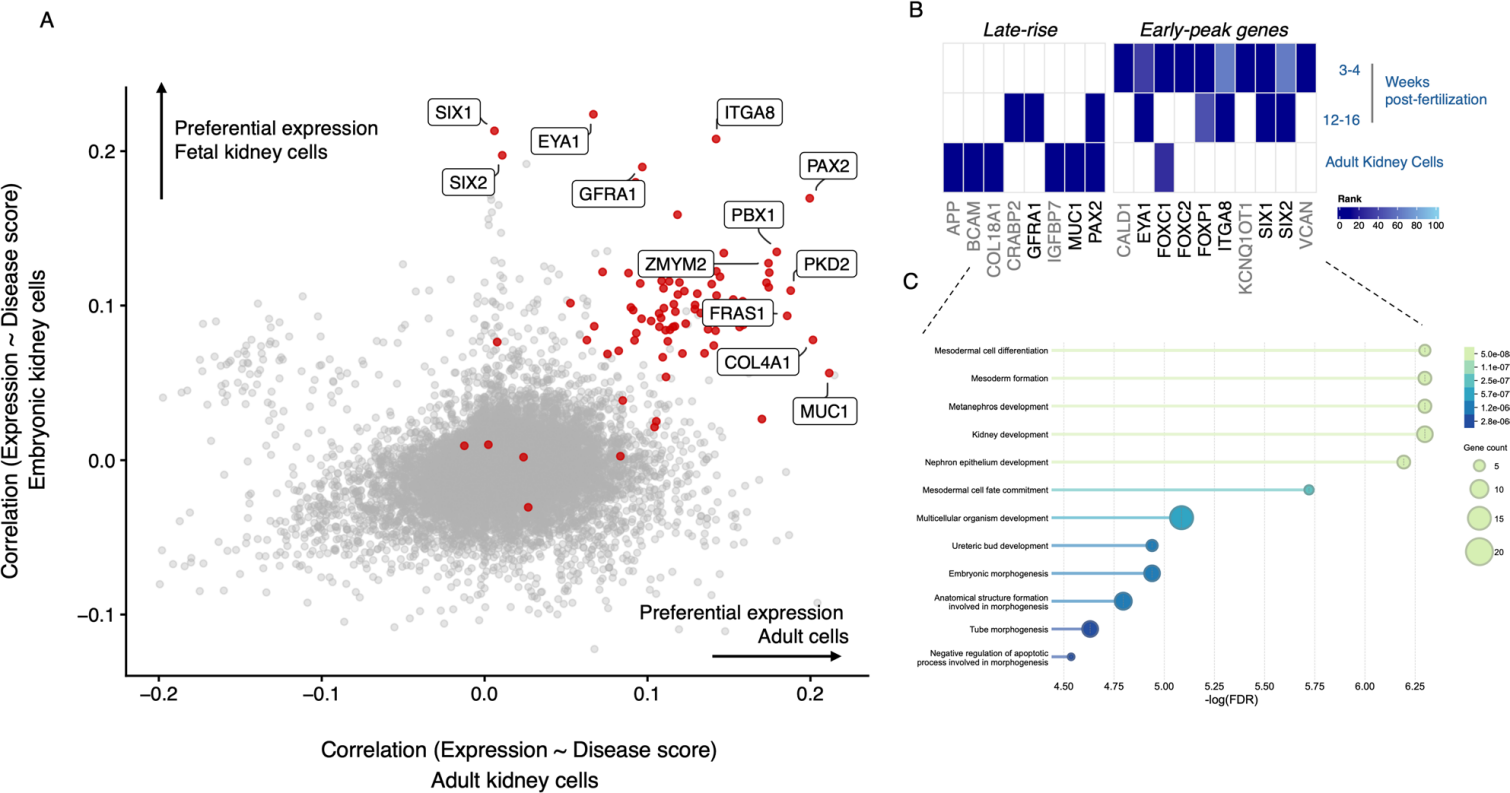
Preferential expression of CAKUT genes in embryonic versus adult kidney cells. (**A**) Each point represents a gene, with its position determined by the correlation between gene expression and disease score (scDRS) in adult kidney cells (x-axis) versus embryonic kidney cells (y-axis). Red points indicate known CAKUT-associated genes. Genes positioned in the upper right quadrant are positively correlated with disease in both embryonic and adult contexts, while those shifted vertically above the diagonal line show preferential expression correlation in embryonic kidney cells. Genes in the lower right quadrant show stronger correlation with disease in adult cells. Labeled red genes (e.g., *EYA1*, *SIX1*, *ITGA8*) are known to play key roles in kidney development and highlight the embryonic specificity of CAKUT-associated gene expression programs. Arrows indicate the direction of preferential expression in either embryonic or adult kidneys. (**B**) Developmental dynamics of CAKUT-associated genes. Heatmap showing temporal expression patterns of representative *late-rise* and *early-peak* CAKUT genes across fetal kidney stages (3–4 and 12–16 weeks post-fertilization) and adult kidney cells. Early-peak genes exhibit strong expression during early nephrogenesis, whereas late-rise genes maintain or increase expression in later stages and adult tissue. (**C**) Functional enrichment of early-peak CAKUT genes. Bubble plot showing Gene Ontology (GO) biological processes enriched among early-peak genes. Bubble size represents the number of genes in each category, and color intensity reflects the significance level (–log₁₀FDR)

To assess whether the CAKUT genes driving preferential expression in adult kidney cells differ from those in fetal cells, we compared the ranking of CAKUT genes across the two datasets (Fig. 3A). Genes that contribute disproportionately to embryonic-specific expression include *EYA1*, *SIX1*, *SIX2*, *ITGA8*, *GFRA1*, *HOXA11L*, *ETV3*, *REN*, and *SALL1*. In contrast, genes such as *MUC1*, *NPNT*, and *COL4A1* show stronger contributions to adult cell-specific expression. Notably, *PAX2* ranks among the top contributors in both embryonic and adult datasets.

An illustrative example is *EYA1*, which encodes a member of the eyes absent (EYA) family of transcriptional coactivators and phosphatases. The protein plays a critical role in kidney development and *EYA1* is one of the genes most frequently implicated in monogenic forms of CAKUT, alongside *PAX2* [7, 37]. Notably, *EYA1*, *SIX1*, *SIX2*, and *ITGA8* were also prioritized in the study by Shierbaum et al. [23] and exhibit temporal co-expression in nephron progenitor cells, supporting their functional relevance during early kidney morphogenesis. In contrast, *MUC1*, which contributes more strongly to adult cell type specific expression, encodes a transmembrane glycoprotein and has been implicated in adult-onset renal pathologies and particularly in autosomal dominant tubulointerstitial kidney disease (ADTKD) [38]. Overall, the separation of embryonic- and adult-associated CAKUT genes in their contribution to cell type specific expression reinforces the developmental specificity of their functional roles. Moreover, the presence of genes like *PAX2*, which show high specificity with both embryonic and adult cell types, underscores their broad importance across developmental stages.

### Developmental stage–specific expression patterns of CAKUT genes

In many congenital disorders, the contribution of disease-associated genes is not constant but arises at specific developmental windows. For example, in our previous work on esophageal atresia, we demonstrated that the expression of *SOX2* is the strongest determinant of single-cell disease scores at embryonic day 3.5 (E3.5), with this correlation declining as the embryo transitions into later stages [34]. Similarly, in congenital heart defects, mutations in *NKX2-5* and *GATA4* act during very early cardiac looping and chamber specification, whereas their influence diminishes once structural formation is complete [39].

Motivated by these precedents, we sought to identify the earliest window at which CAKUT-associated genes leave a detectable imprint. To this end, we analyzed the human embryonic single-cell atlas from Xu et al. [40], which profiles ~ 180,000 cells spanning gestational weeks 4–6 (Carnegie stages 12–16). We observed a significant preferential expression of CAKUT genes across multiple mesodermal derivatives, including intermediate mesoderm (IM-1, IM-2), somites, and head mesoderm. These findings indicate that preferential expression of CAKUT genes can already be detected in mesodermal progenitor populations as early as 3–4 weeks post-fertilization, prior to the formation of the definitive metanephric kidney, which begins development around week 5. To determine which specific CAKUT genes are most prominent early on versus those that gain importance later, we next compared their preferential expression across embryonic, fetal, and adult kidney datasets.

We particularly examined the developmental timing of gene expression specificity by comparing CAKUT gene enrichment across three distinct time points: (i) embryonic kidney data from the atlas of Xu et al. (corresponding to the Carnegie stages of CS12–CS16), (ii) fetal kidney cells from Hochane et al. [27], and (iii) adult kidney data from Lake et al. [28]. Our goal was to identify genes that exhibit high cell-type specificity early in development but diminish in prominence at later stages. We focused on genes ranked among the top 5 in cell-type specificity at our earliest time point (CS12-CS16) that fell substantially in rank in the later fetal stage as well as adult cells.

This analysis yielded a refined set of early-peak genes, including *APP*, *BCAM*, *COL18A1*, *CRABP2*, *GFRA1*, *IGFBP7*, *MUC1*, *PAX2*, *CALD1*, *EYA1*, *FOXC1*, *FOXC2*, *FOXP1*, *ITGA8*, *KCNQ1OT1*, *SIX1*, SIX2, and *VCAN*, that exhibited strong enrichment at early developmental stages (Carnegie stage 12) but progressively declined in prominence at later stages (Fig. 3B). Notably, transcriptional regulators such as *SIX1*, *SIX2*, *FOXC1/2*, *EYA1*, and *PAX2* are directly involved in the GDNF–RET signaling axis that governs ureteric bud induction and branching morphogenesis. In parallel, several enriched genes—including *COL18A1*, *ITGA8*, *VCAN*, and *CALD1*—encode components of the extracellular matrix (ECM) and adhesion pathways, underscoring the role of ECM remodeling in establishing a permissive mesenchymal environment for ureteric bud invasion. Importantly, temporal expression profiles and GO enrichment analyses (Fig. 3C) indicate that these early-peak genes act primarily within mesonephric and mesodermal compartments, suggesting that CAKUT-associated mutations in these genes may perturb mesodermal lineage programs that prefigure and scaffold the onset of ureteric bud development. We note, however, that these inferences should be interpreted with caution, as the datasets analyzed derive from independent studies across developmental stages rather than from direct longitudinal profiling.

### Multi-organ and cross-tissue expression patterns of CAKUT genes

CAKUT encompasses a broad spectrum of developmental malformations that extend beyond the kidney, frequently affecting other components of the urinary tract such as the ureter and bladder. For example, ureteropelvic junction obstruction, vesicoureteral reflux, megaureter, and bladder exstrophy are all part of the CAKUT spectrum and reflect developmental defects outside the kidney itself [1, 2, 25]. These conditions can occur in isolation or alongside renal malformations, underscoring the importance of examining CAKUT gene expression beyond kidney cells.

To explore whether CAKUT-associated genes exhibit organ-specific expression patterns, we analyzed single-cell transcriptomic data from the adult ureter and bladder and compared the results to adult kidney data. In the ureter, we observed the strongest association in smooth muscle cells (*p* = 0.00099) and fibroblasts (*p* = 0.00099), including both general and specific subtypes. In the bladder, CAKUT gene expression was most enriched in peri-urothelial fibroblasts (*p* = 0.00599), general smooth muscle cells (*p* = 0.0079), and intra-muscular fibroblasts (*p* = 0.0079). These findings parallel those in the adult kidney, where mural cells, collecting duct principal cells, and vascular-associated smooth muscle cells showed the most significant associations (Table 1). Across all three organs, CAKUT-associated genes were consistently enriched in stromal and mesenchymal cell types, highlighting a shared cellular context that may underlie disease pathogenesis.

Because the analyzed datasets originate from independent studies with different cell-type annotation schemes, we sought to ensure that our findings were not driven by dataset-specific biases. To this end, we repeated the analysis using the harmonized *Tabula Sapiens* atlas [31]. This large-scale reference atlas comprises over 1.1 million cells across 28 organs from 24 healthy human donors and is processed using a unified computational pipeline with standardized cell-type annotations based on a shared ontology. We restricted the analysis to the kidney and bladder cells that were available in Tabula Sapiens atlas. We observed a high degree of concordance with our initial results, with CAKUT genes showing preferential expression in endothelial and kidney epithelial populations, as well as in fibroblast and myofibroblast populations in the bladder (Table S7). These findings support the robustness of our results across independently generated datasets and consistent annotation frameworks.

**Fig. 4:**
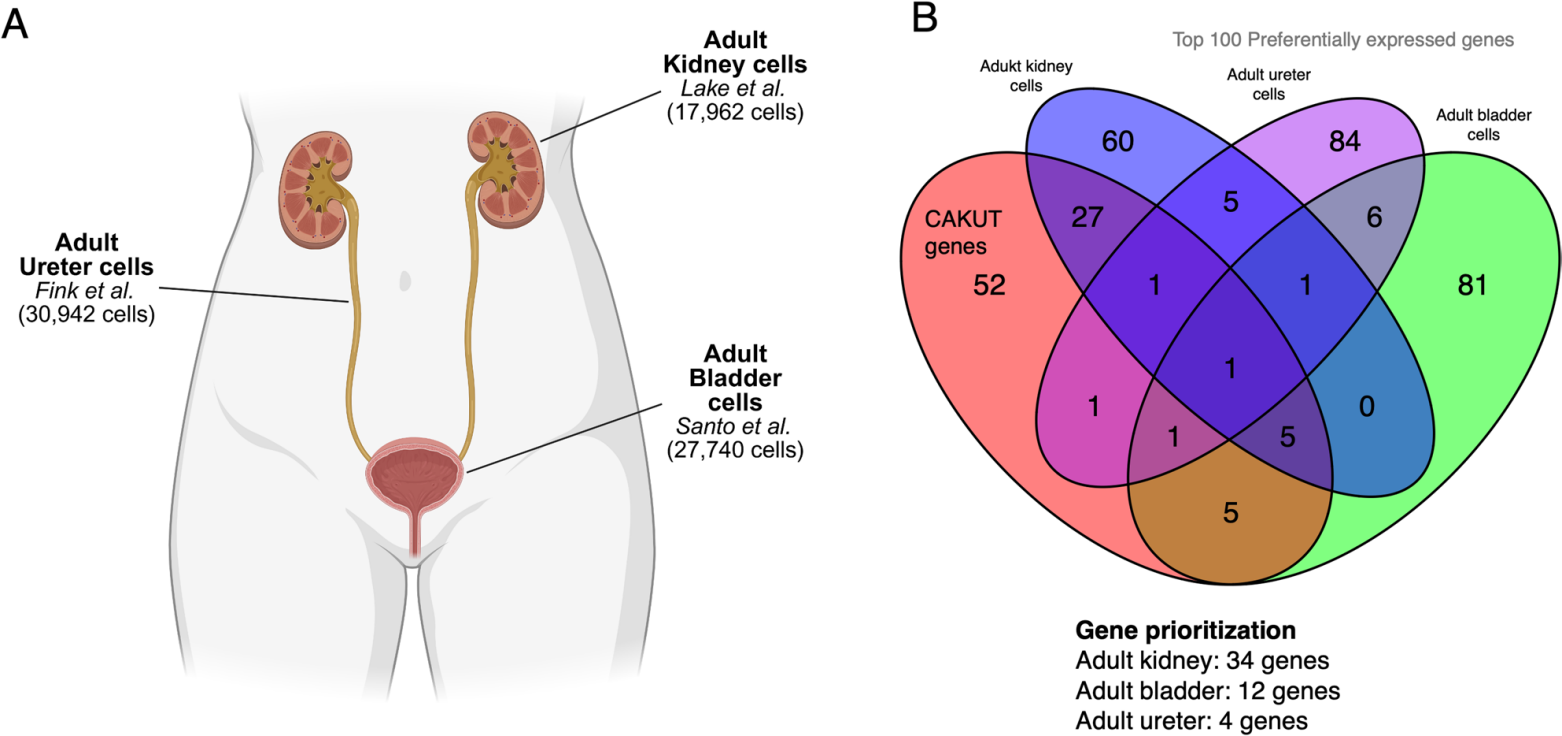
Gene prioritization across adult urinary tract cell types. A) Venn diagram showing the overlap between CAKUT genes and the top 100 preferentially expressed genes in adult kidney (Lake et al. [28], 17,962 cells), adult ureter (Fink et al. [29], 30,942 cells), and adult bladder (Santo et al. [30]; 27,740 cells). The overlap reveals both shared and organ-specific gene expression patterns. B) Gene prioritization results based on overlap with CAKUT genes: 34 genes were identified as kidney-specific, 12 genes as bladder-specific, and 4 genes as ureter-specific CAKUT-related candidates.Created in BioRender. K, V. (2026) https://BioRender.com/a4r2gmh.

To prioritize our initial list of CAKUT genes according to organ-specific relevance, we selected the top 100 genes with the highest cell type–specific expression scores in each organ (Fig. 4). Kidney cells possessed a disproportionately high number of CAKUT genes, with 33 overlapping hits in the adult kidney dataset. Among these, *MUC1* (ENSG00000185499) ranked highest, appearing as the third most cell type–specific gene overall. By contrast, only four overlaps were detected in the ureter dataset, with *COL4A1* (ENSG00000187498) as the top-ranked gene, and 12 overlaps were observed in the bladder dataset, where *BNC2* (ENSG00000173068) ranked highest. Notably, *COL4A1* was the only CAKUT gene consistently represented among the top cell type–specific gene sets across kidney, ureter, and bladder. This pattern underscores both the dominant contribution of kidney-enriched genes to CAKUT risk and the unique cross-organ role of *COL4A1* in maintaining the structural and functional integrity of the urinary tract.

## Discussion

In this study, we used single-cell transcriptomic data from human kidney, ureter, and bladder to systematically map the cell types with preferential expression of genes associated with congenital anomalies of the kidney and urinary tract (CAKUT). Our analysis revealed that canonical CAKUT genes—including *HOXA11*,* EYA1*,* PAX2*,* FOXC1*,* HNF1B*,* GATA3*,* SIX1*, and *ITGA8*—are strongly expressed in fetal mesangial and nephron tubule epithelial cells, as well as in postnatal populations of mural cells, fibroblasts, and smooth muscle cells across urinary tract tissues. These patterns point to a predominantly mesenchymal origin of CAKUT gene expression. This is consistent with developmental models in which *FOXC1/FOXC2*,* ROBO1/2*,* PAX2*,* HOXA11*, and *EYA1* orchestrate mesenchymal signaling required for ureteric bud induction [2]. Together, our findings reinforce the view that CAKUT genes cluster in mesenchymal and fibroblast lineages, underlining the central role of mesenchymal regulation in the pathogenesis of urinary tract malformations.

A striking result from our embryonic single-cell analysis was the early enrichment of genes involved in collagen biology and extracellular matrix (ECM) organization, including *COL1A2*,* COL2A1*,* COL3A1*,* COL4A1*,* PCOLCE*,* ITGA8*,* VCAN*, and *CALD1*. These genes peaked at Carnegie stage 12 and declined thereafter, suggesting that ECM remodeling and collagen fibril organization represent some of the earliest molecular processes likely perturbed in CAKUT. This observation aligns with and extends prior multi-omics studies of amniotic fluid from CAKUT pregnancies, which consistently identified collagen fragments and ECM pathways, with PI3K–AKT signaling emerging as a dominant axis [41, 42]. Genetic data also converge on this mechanism: *COL4A1* mutations were first described in HANAC syndrome [43] and later recognized as a potential cause of non-syndromic CAKUT [44], while *COL4A3–5* variants recur across nephrological cohorts [45]. Within this broader ECM signature, COL4A1 is the only CAKUT gene showing enrichment across kidney, ureter, and bladder tissues in our analysis, encodes the α1 chain of type IV collagen, a major structural component of basement membranes. This observation is also consistent with recent studies showing that specific collagen peptides detected in urine are associated with the prognosis of chronic kidney disease in adults and reflect the degree of kidney fibrosis in situ [46]. Furthermore, recent peptidomic analyses of amniotic fluid have identified peptide signatures composed largely of fragments derived from extracellular matrix proteins, including collagens, suggesting that extracellular matrix remodeling is a detectable feature of fetal kidney development and pathology [42, 47]. Together, these findings support the idea that alterations in extracellular matrix components such as *COL4A1* may influence multiple stages of urinary tract development and contribute to the diverse phenotypic manifestations observed in CAKUT.

Among the genes prioritized by our scDRS analysis, *CRABP2* and *CITED1* ranked among the highest-scoring candidates, highlighting them as potentially relevant to CAKUT biology. *CRABP2* (Cellular Retinoic Acid Binding Protein 2) is involved in intracellular retinoic acid signaling, a pathway that plays a critical role in kidney and urinary tract development [35]. Retinoic acid regulates organ development through transcription activation or repression and is required for ureter maturation and ureter–bladder connectivity [48, 49]. *CITED1* (CBP/p300-interacting transactivator with ED-rich tail 1) is well known as a marker of nephron progenitor cells within the cap mesenchyme of the developing kidney [50] and has been implicated in the transcriptional regulation of progenitor cell maintenance and differentiation during nephrogenesis [51]. These findings suggest that *CRABP2* and *CITED1* mark nephron progenitor–associated transcriptional programs that are enriched in CAKUT-relevant cellular contexts. We acknowledge that the deletion of CITED1 or CRABP2 in mice [52, 53] has been reported to result in no overt renal phenotypic defects which may suggest potential functional redundancy within these developmental programs. However, their high scDRS ranking remains relevant as it identifies them as core components of the transcriptional network characterizing the disease-relevant cell state. While this observation should not be interpreted as evidence of direct causality, it highlights gene modules and cellular states that warrant further investigation in the context of kidney development and disease.

More generally, our approach builds on a growing framework for identifying disease-relevant cell types by integrating genetic association data with single-cell transcriptomics [17, 20, 54]. The central premise is that genetic studies provide sets of genes linked to disease risk, but do not resolve the cellular contexts or developmental stages in which these genes act. Single-cell RNA-seq data offer a complementary layer of information, enabling the mapping of disease-associated gene signals onto specific cell types and states. Methods such as scDRS [17], which we employed here, assess whether genes associated with a given disease show coordinated enrichment within particular cellular populations by comparing their expression to matched control gene sets. In this context, the prioritization of genes such as *CITED1*and *CRABP2* arises not from their direct genetic association with CAKUT, but from their strong co-expression with CAKUT genes in disease-relevant cell states, particularly nephron progenitor populations. This distinction is important, as it emphasizes that scDRS identifies transcriptional programs and cellular contexts linked to diseases such as CAKUT. Complementary approaches, such as regression-based methods that relate gene-level association statistics to cell type–specific expression (e.g., Seismic [55]), can provide further evidence when sufficiently powered GWAS are available, and represent a promising direction for future CAKUT studies.

Although CAKUT is clinically defined by urinary tract malformations, the phenotypic spectrum often extends beyond the kidney. Up to half of patients, especially in pediatric autopsy cohorts, present with anomalies in other organ systems, including cardiovascular, gastrointestinal, and central nervous system malformations, as well as craniofacial, auditory, skeletal, pulmonary, and neurodevelopmental abnormalities [2]. These associations suggest that CAKUT frequently reflects a systemic disruption of developmental programs rather than an isolated urogenital defect. Our single-cell analysis supports this broader view: while CAKUT genes were most enriched in mesodermal derivatives relevant to urinary tract development (intermediate mesoderm, metanephric mesenchyme, ureteric bud), we also observed expression in embryonic lineages not traditionally linked to the urinary tract, including craniofacial mesenchyme, somites, head mesoderm, and neural crest–derived populations. Although some of these signals may reflect noise, the overlap with the pattern of comorbidities in CAKUT patients argues that they might capture pleiotropic effects of CAKUT genes.

We also emphasize that investigating the cellular context underlying the emergence of congenital diseases such as CAKUT will benefit from the rapidly growing body of single-cell and single-cell multiomic studies across urinary tract tissues, including the ureter and bladder. Recent work has begun to generate high-resolution atlases of these tissues, complementing kidney-focused datasets and enabling a more comprehensive view of urinary tract development and disease (e.g., Fink et al. [29]; Lee et al. [56]; Li et al. [57]), and in cross-species analysis between human and mouse (Yu et al. [58]). Integrating these resources, either through joint analysis or computational meta-analysis using established frameworks will enable the reconstruction of spatial and temporal gene expression programs across the entire urogenital system. Such integrative approaches will be essential to pinpoint the precise developmental timing, cellular origins, and molecular mechanisms underlying CAKUT, and may ultimately guide the development of more targeted diagnostic and therapeutic strategies.

Our findings inform the debate regarding the utility of the “CAKUT” umbrella term. While the category encompasses a wide variety of anatomical anomalies, our results provide evidence that CAKUT genes show enriched expression across overlapping sets of mesenchymal and stromal cell types in the kidney, ureter, and bladder. These patterns indicate that, despite outward clinical heterogeneity, CAKUT genes may participate in shared developmental contexts, particularly those involving epithelial–mesenchymal interfaces and extracellular matrix (ECM) organization. We note, however, that our results reflect expression enrichment rather than direct evidence of developmental perturbation, and further studies will be required to clarify the contribution of different cell types to CAKUT pathogenesis. Thus, the CAKUT designation remains useful not only as a clinical grouping but also as a framework for studying the developmental pathways and cellular contexts associated with these congenital anomalies.

This study has several limitations. First, our analyses rely on publicly available single-cell datasets, which often include limited sample sizes and incomplete metadata regarding demographic and clinical variables such as ethnicity, sex, and detailed phenotypic information. As a result, it was not possible to systematically assess how these factors may influence cell-type–specific expression patterns of CAKUT genes. Second, public datasets are generated using different experimental protocols and sequencing platforms, which may introduce technical variability or batch effects. To mitigate this concern, we performed analyses independently within each dataset rather than integrating them into a shared embedding, thereby reducing the likelihood that cross-study batch effects drive the enrichment signals observed. Finally, while transcriptomic enrichment provides insight into the cellular contexts in which disease genes are preferentially expressed, such analyses do not establish causal mechanisms. Future studies combining larger, harmonized datasets and functional experiments will be important to further refine the cellular and molecular mechanisms underlying CAKUT.

## Supplementary Information

Below is the link to the electronic supplementary material.

Supplementary Material 1

## Data Availability

All primary single-cell datasets analyzed in this study are publicly available with no access restrictions. Accession numbers and permanent dataset identifiers include CELLxGENE collections bcb61471-2a44-4d00-a0af-ff085512674c, 981b0c87-76d0-4edf-b201 58c52b5cd2f0, 0e54d4de-44f0-4d50-8649-b5c2bbe8f5d1, and af286675-7167-4816-a907-c72d41b73d37, as well as the Gene Expression Omnibus (GEO) dataset GSE157329. Full dataset accessibility, direct links, and processing details are provided on the project GitHub repository (https://github.com/dasmeh/CAKUT). No controlled-access datasets were used, and no additional permissions were required to access the data.
